# Relationship between Neuroglial Apoptosis and Neuroinflammation in the Epileptic Focus of the Brain and in the Blood of Patients with Drug-Resistant Epilepsy

**DOI:** 10.3390/ijms232012561

**Published:** 2022-10-19

**Authors:** Tatiana V. Sokolova, Yulia M. Zabrodskaya, Anastasia V. Litovchenko, Natalia M. Paramonova, Vugar R. Kasumov, Svetlana V. Kravtsova, Ekaterina N. Skiteva, Daria A. Sitovskaya, Elena D. Bazhanova

**Affiliations:** 1 Polenov Neurosurgical Institute—Branch of the Almazov National Medical Centre, 197341 St. Petersburg, Russia; 2 Sechenov Institute of Evolutionary Physiology and Biochemistry, Russian Academy of Sciences, 194223 St. Petersburg, Russia; 3 Department of Neurology, St. Petersburg State Pediatric Medical University, 194100 St. Petersburg, Russia; 4 State Scientific Center of the Russian Federation, Institute of Biomedical Problems of the Russian Academy of Sciences, 123007 Moscow, Russia; 5 Golikov Research Center of Toxicology, 192019 St. Petersburg, Russia; 6 Laboratory of Apoptosis Investigation, Astrakhan State University, 414040 Astrakhan, Russia

**Keywords:** drug-resistant epilepsy, apoptosis, neuroinflammation, cytokines, neurons, glia

## Abstract

Neuroglial apoptosis and neuroinflammation play an important role in epileptogenesis. The aim of this study is to evaluate neuronal and glial apoptosis in association with neuroinflammation in brain epileptic focus and inflammatory changes in blood in patients with focal drug-resistant epilepsy (DRE). Pathological changes in the temporal lobe in epilepsy (histology, transmission electron microscopy), levels of apoptotic and neuroinflammatory proteins: active caspase-3 (immunohistochemistry), full-length form caspase-3, caspase-9, FAS, FAS-L, NF-kB, TNF-α, p53 (Western blot), and cytokine levels in blood: IL-1β, IL-2, IL-4, IL-7, TNF-α, etc. (multiplex analysis) were studied. In the present work, ultrastructural and immunohistochemical apoptotic signs were found in neurons and oligodendrocytes in the temporal lobe of DRE patients. Levels of proinflammatory cytokines that play a role in apoptosis (TNF-α, FAS, NF-kB) were increased. The blood concentration of IL-4, IL-7, TNF-α is increased and IL-2 is reduced. Oligodendroglial apoptosis has been shown to play an important role in DRE pathogenesis and to explain demyelination. Thus, a comprehensive analysis of revealed changes in the blood and brain in DRE patients showed the neuroinflammation in the epileptic focus, which was combined with the development of apoptosis of glial cells and neurons. This creates conditions for the development of drug resistance and the epilepsy progression.

## 1. Introduction

Drug-resistant epilepsy (DRE) is characterized by spontaneously recurring seizures, cognitive deficit, and brain structural changes. Status epilepticus is a prolonged seizure that damages neurons and neural networks; its appearance precedes drug resistance [1,2]. Although more than 30 anti-seizure medications (ASM) are currently available [3], unfortunately, long-term regular ASM intake allows only 40–60% of patients to achieve remission [4]. Surgical correction is an effective method for DRE treatment, however the positive results of surgery are noted only in 55–74% of patients [5,6]. Thus, it is important to search for new epileptogenic mechanisms that can become the basis for effective treatment.

Apoptosis, oxidative stress, the loss of inhibitory or excitatory neurons, and inflammation play a key role in epilepsy progression [7,8,9]. We suggest that since the neuronal and glial components are closely related, it is necessary to study the functioning of both neurons and glia in epilepsy [10]. Reactive gliosis and inflammation accompanying neuronal damage contribute to the emergence of hyperexcitability focus [11]. Astrocytes may play a central role in epileptogenesis [4]. The epileptic seizures may be associated with damage to the myelin sheath, which directly affects neuron survival [12]. In this regard, the glia is considered to be a promising new target for alternative ASM [13]. Some studies are devoted to glial apoptosis in epilepsy [14,15], but the main attention is focused on seizure-induced neuronal death [11,16].

The extrinsic (TNF-α, FAS) [17] or mitochondrial apoptotic pathways [18] and the important gene *p53* [19] can play a significant role in epileptogenesis. It is also important to study the caspase activation in epilepsy, which plays a decisive role in apoptosis. Caspase-3 is an effector caspase that activates the endonuclease CAD (caspase-activated DNase), causing DNA breaks in the internucleosomal regions and leading to the destruction and death of cells [10,14,16,19].

Neuroinflammation, closely associated with cell death, is regulated mainly by cytokines. The cytokine system can modulate the functional activity of immunocompetent cells and have cytotoxic and proapoptotic effects. Cytokines can migrate between blood and brain in both directions. The cytokines activate monocytes/macrophages, etc., and together contribute to apoptosis [20]. In this regard, this work aims to evaluate the manifestations of neuronal and glial apoptosis in conjunction with neuroinflammation in the epileptic focus area and inflammatory changes in the blood of patients with DRE.

## 2. Results

### 2.1. Histological Examination

**Figure 1:**
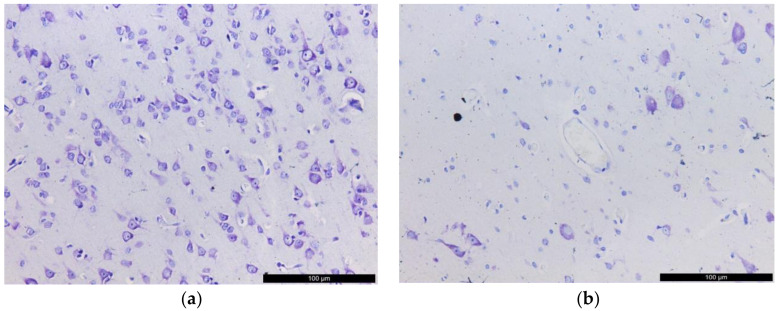

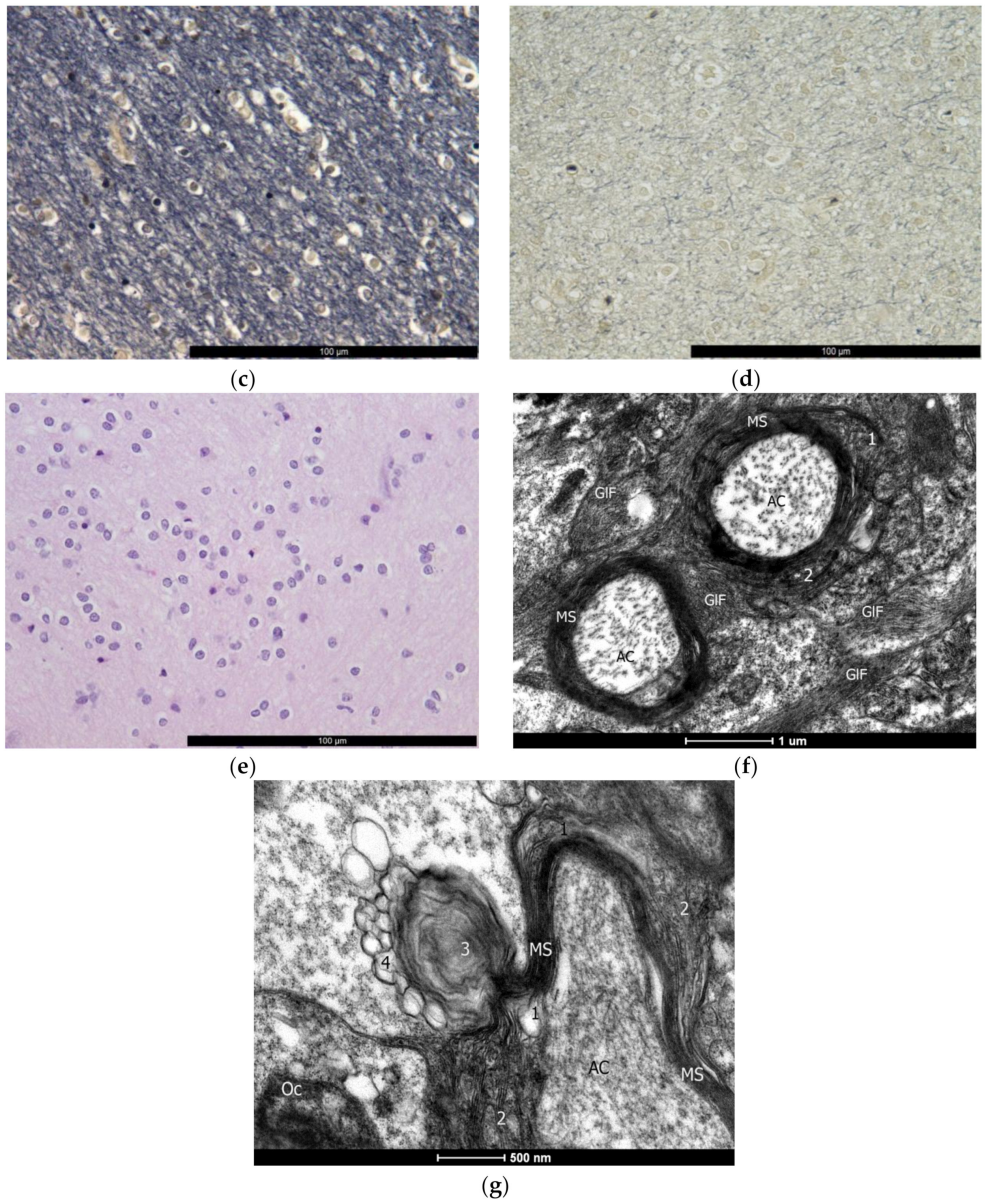
Structural changes in the epileptic focus in the temporal lobe. Light microscopy: (**a**) neurons in the cerebral cortex in the comparison group; (**b**) loss of neurons in the cerebral cortex in a patient with DRE (Nissl staining, 200×); (**c**) white matter rich in myelin in the comparison group; (**d**) white matter demyelination in a patient with DRE (Spielmeier staining, 200×); (**e**) white matter, gliosis (HE, 400×). TEM: (f) destruction of myelin sheaths; bar, 1 μm; 16,500×; (**g**) destruction of myelin sheaths; bar, 500 nm; 26,500×. 1, areas of lamella rupture; 2, delamination of the sheath; 3, myelin dissociation; 4, vesicular disintegration; 5, grainy myelin disintegration; GlF, gliofibrils; Oc, oligodendrocyte; AC, axial cylinder, axon; MS, myelin sheath.

When staining according to Nissl, in all DRE patients, we have seen foci of neuronal loss (Figure 1a,b) and reactive-destructive changes in neurons in the epileptic focus in the temporal lobe. Hydropic dystrophy with chromatolysis and vacuolization of the neuron cytoplasm was oberved, as were hyperchromic shriveled ischemic cells. These changes were accompanied by moderate satelliteitis and neuronophagia, and mild gliosis. In the white matter, cellular gliosis was accompanied by myelin loss (demyelination), as well as damage to the myelin sheaths (Spielmeir staining) (Figure 1d,e), TEM (see below) (Figure 1f,g).

### 2.2. IHC (Active Caspase-3)

To assess the severity of apoptosis in the cortex and in the white matter in the epileptic focus, IHC with caspase-3 (active form) was performed.

**Figure 2:**
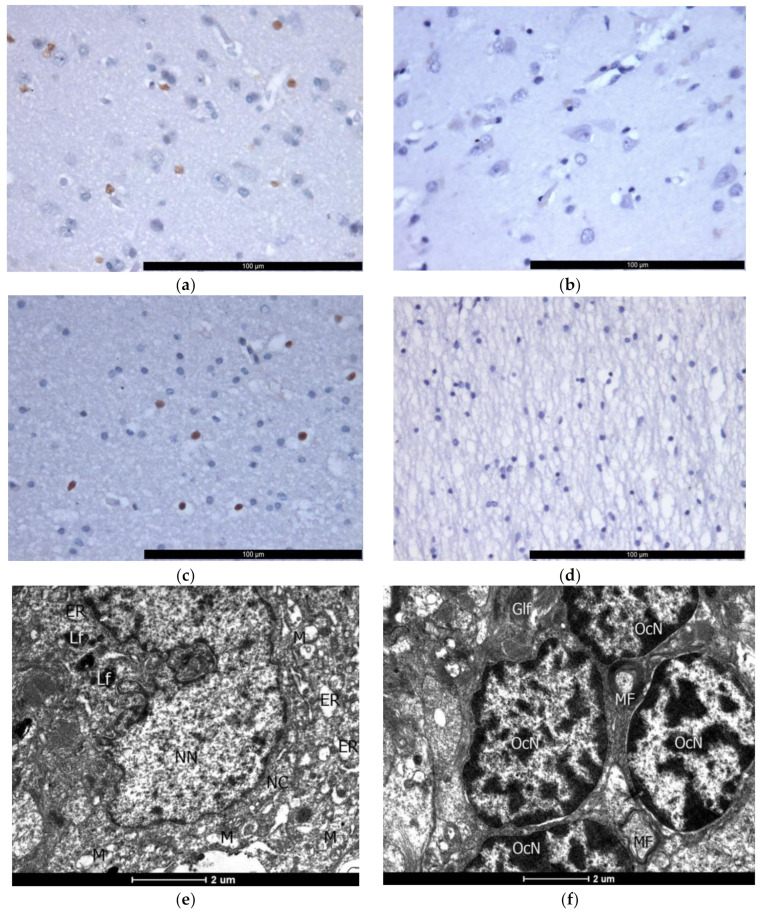
Study of cell apoptosis in the epileptic focus of the temporal lobe. Light microscopy, IHC study of cell apoptosis in the epileptic focus of the temporal lobe: (**a**) the presence of active caspase-3 in glial cells in the cortex of patients with DRE (brown staining in the cell nucleus); (**b**) absence of the active caspase-3 in the cortex cells of patients in the comparison group (without epilepsy); (**c**) the presence of active caspase-3 in glial cells in the white matter of patients with DRE (brown staining in the cell nucleus); (**d**) absence of the active caspase-3 in the white matter cells of patients in the comparison group (without epilepsy), 400×. TEM, the initial stage of apoptosis: (**e**) cortical neuron with an elongated, curved nucleus (NN, neuron nucleus), finely condensed chromatin fragments in the karyoplasm, destructively altered mitochondria (M) in the cytoplasm (NC, neuron cytoplasm), dilated tubules of the endoplasmic reticulum (ER) and lipofuscin granules (LF); bar, 2 μm; (**f**) an accumulation of oligodendrocytes in the white matter of the brain, the heterochromatin of which is distributed throughout the nucleus (OcN, oligodendrocyte nucleus) in large conglomerates. GlF, glyofibrils; MF, myelin fibers; bar, 2 μm; 8200×.

In all DRE patients, we have seen the presence of active caspase 3 in the nuclei of glial cells of the gray and white matter of the brain. In the cortex, the distribution of immunoreactive gliocytes was diffuse, predominantly uniform in all cortical layers, and nuclear staining was intense. In 20% of cases, the presence of active caspase-3 was observed in separate nuclei of cortical neurons; in 80% of cases, active caspase-3 was absent in neurons. In comparison, for the group in the cortex, in 66.7% of cases, active caspase-3 was absent in glial cells and neurons (Figure 2a,b, Table 1); in 33.3% of cases, we observed single stained nuclei of gliocytes. In the white matter, immunoreactive gliocytes were also diffusely distributed, predominantly evenly, and the staining of the nuclei was less intense than in the cortex. While in the comparison group, the active form of caspase-3 was absent in the white matter (Figure 2c,d, Table 1).

### 2.3. Ultrastructural (TEM) and Histological Examination of Apoptosis in Epileptic Focus

**Figure 3:**
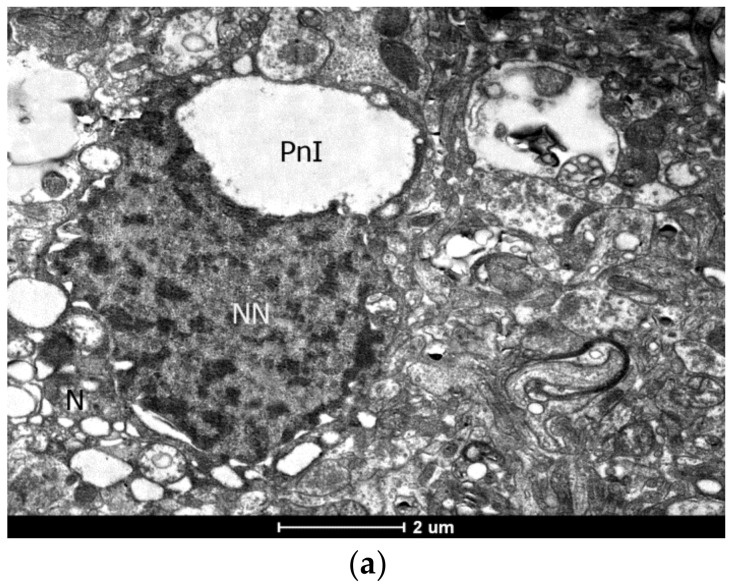

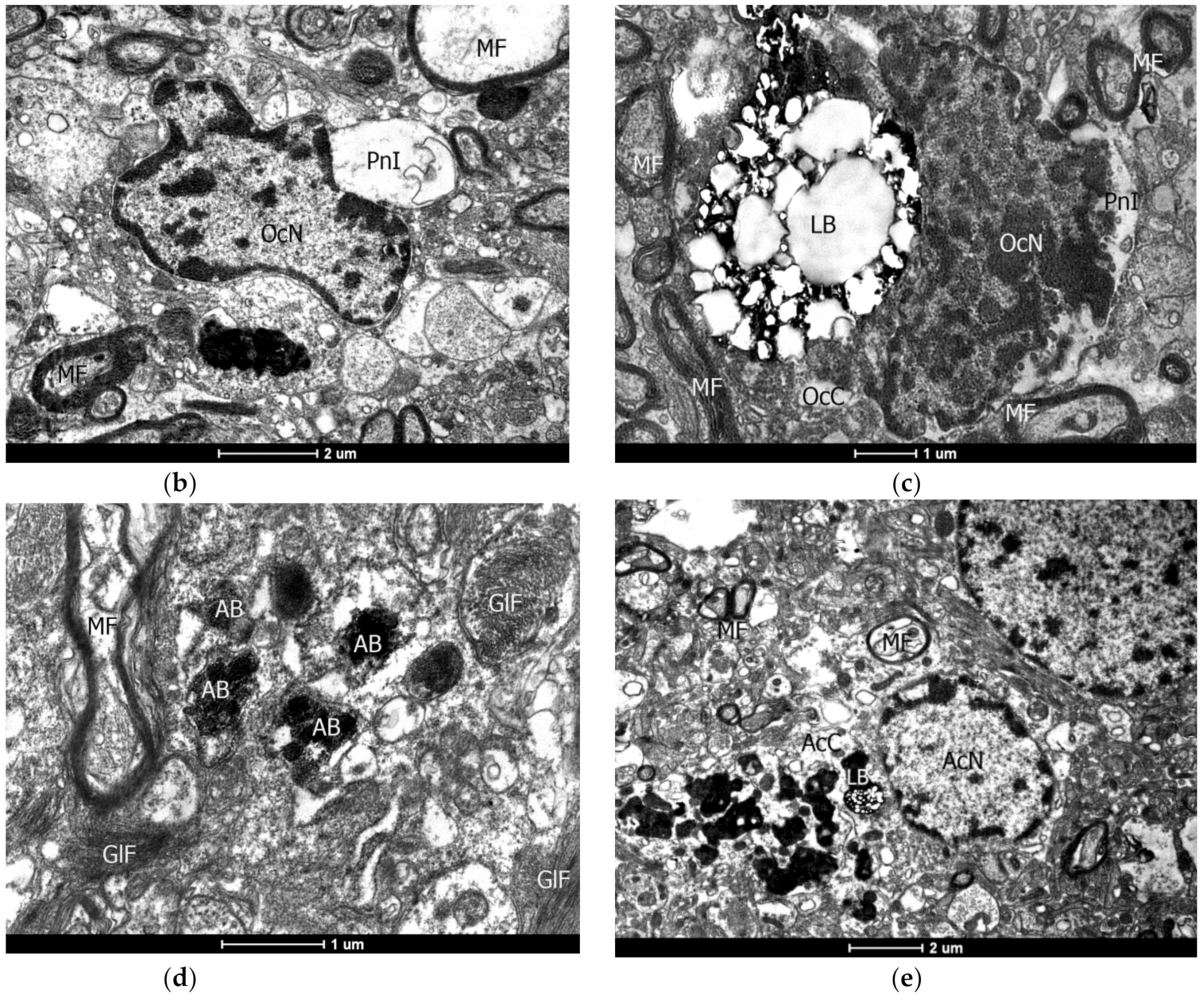
Apoptosis of neurons and oligodendroglia in the cerebral cortex with the formation of perinuclear inflation (PnI). TEM: (**a**) vacuoles and perinuclear inflation in an apoptotic neuron (N); bar, 2 μm; (**b**) perinuclear inflation in the oligodendrocyte; bar, 2 μm; 8200×; (**c**) apoptosis of oligodendrocyte at the stage of nuclear disintegration with a Lewy body (LB) in the cytoplasm (OcC), on the side of the rupture of the nuclear envelope has many fragments of nuclear matter. MF, myelin fiber; NN, neuron nucleus; OcN, oligodendrocyte nucleus; PnI, perinuclear inflation; bar, 1 μm; 9900×.; (**d**,**e**) formation of apoptotic bodies (AB) and their utilization: (**d**) apoptotic bodies in the intercellular space; bar, 1 μm; 16,500×; (**e**) astrocyte (Ac), performing the function of a macrophage, with a large accumulation of cellular degradation products, lipofuscin granules (Lf) and Lewy body (LB) in the astrocyte cytoplasm (AcC); AcN, astrocyte nucleus; Glf,glyofibrils; MF, myelin fiber; bar, 2 μm; 6000×.

The IHC study has shown that predominantly glia, not neurons, undergo apoptosis. TEM has revealed that in the cortex of DRE patients, a significant number of neurons have shown morphological signs of apoptosis (Figure 2e, Figure 3a). We have observed different apoptotic stage neurons and glial cells; early signs of DNA fragmentation based on the presence of heterochromatin clumps spread throughout the karyoplasm (Figure 2e,f). At the later apoptosis stages, we have observed nuclear disintegration against the background of irreversible degenerative changes in the cytoplasm, pronounced perinuclear pockets, sharply dilated tubules of the endoplasmic reticulum and cisterns of Golgi apparatus, which often combined into huge vacuoles (Figure 3).

Most cortical oligodendrocytes and almost all oligodendrocytes in the white matter have shown signs of apoptotic destruction. The nuclei were irregular, angular shaped, and their karyoplasm was filled with many dense heterochromatin lumps of different sizes. Often, the сytoplasm of neurons was hypertrophied, with a compacted matrix, sharply vacuolated organelles, and homogeneous condensation of karyoplasm, which is rather characteristic of necrotic cell death. Single apoptotic astrocytes were also observed. Thus, we show that neuronal damage, both necrotic and apoptotic, is recorded in the epileptic focus. In the gliocytes, apoptosis is more pronounced in oligodendrocytes (Figure 3b,c).

We have also observed the stages of cell disintegration into apoptotic bodies as a result of disruption of the cell membrane integrity. Astrocytes often act as macrophages capable of phagocytosis of apoptotic bodies (Figure 3d,e). In addition, TEM has revealed a wide variety of pathological signs of destruction in the structure of the myelin sheath of axons (Figure 1f,g).

### 2.4. WB

#### 2.4.1. WB. Epileptic Focus

**Figure 4:**
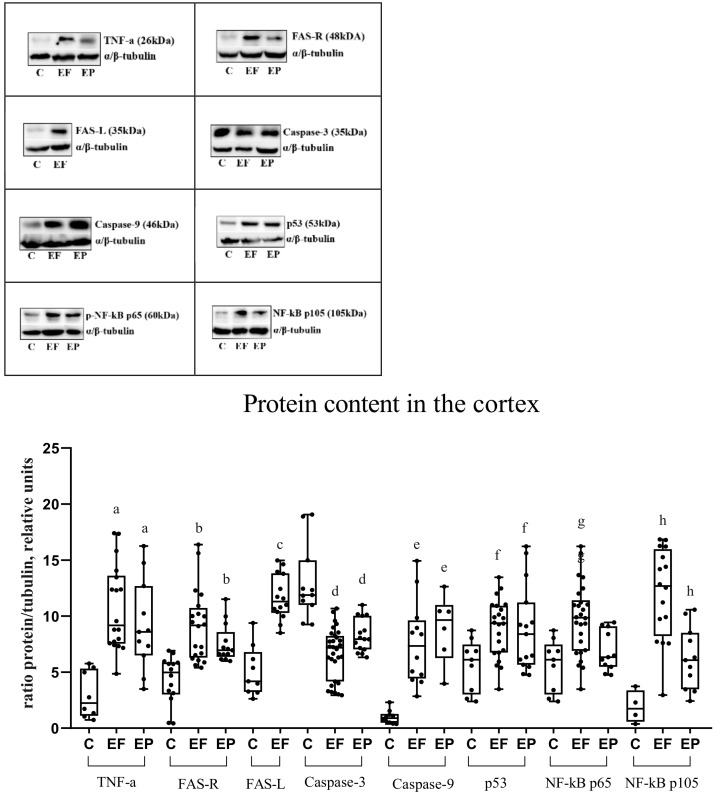
Content of TNF-α, FAS-R, FAS-L, caspase-3, caspase-9, p53, p-NF-kB p65, p-NF-kB p105 in the temporal lobe cortex. Lysates of biopsies of the human temporal lobe cortex were studied on a 10% polyacrylamide gel using Western blot. C, control group; EF, epileptic focus; EP, perifocal zone. Significant differences: a, differences in the content of TNF-α protein in the cortex compared to the control group, statistically significant at *p* < 0.05; b, differences in the content of FAS-R protein in the cortex in comparison with the control group, statistically significant at *p* < 0.05; c, differences in the content of FAS-L protein in the cortex compared to the control group, statistically significant at *p* < 0.05; d, differences in the content of caspase-3 protein in the cortex compared with the control group, statistically significant at *p* < 0.05; e, differences in the content of caspase-9 protein in the cortex compared to the control group, statistically significant at *p* < 0.05; f, differences in the content of p53 protein in the cortex in comparison with the control group, statistically significant at *p* < 0.05; g, differences in the content of p-NF-kB p65 protein in the cortex compared to the control group, statistically significant at *p* < 0.05; h, differences in the content of NF-kB p105 protein in the cortex compared to the control group, statistically significant at *p* < 0.05.

**Figure 5:**
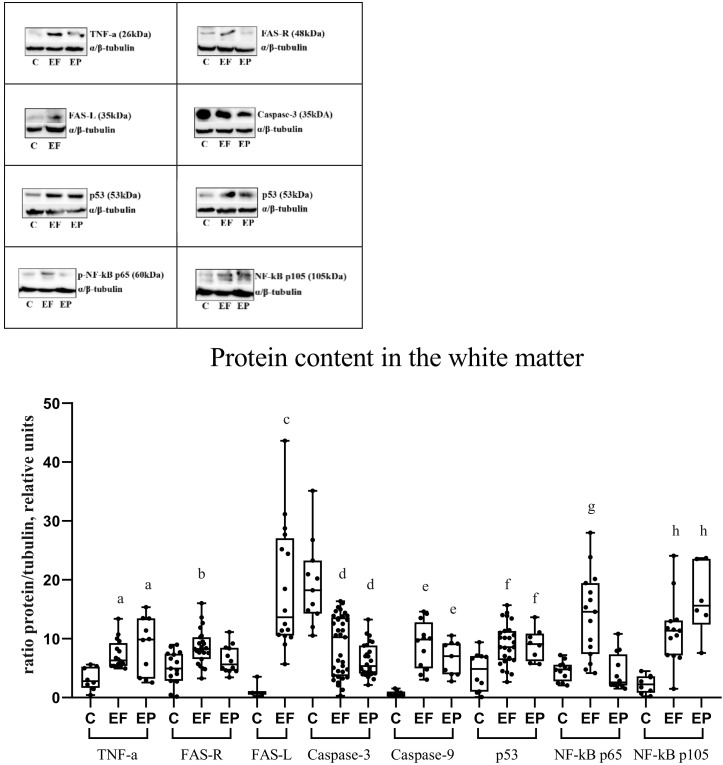
Content of TNF-α, FAS-R, FAS-L, caspase-3, caspase-9, p53, p-NF-kB p65, p-NF-kB p105 in the white matter of the temporal lobe of the brain. Lysates of human white matter biopsy were studied on a 10% polyacrylamide gel by Western blot. C, control group; EF, epileptic focus; EP, perifocal zone. Significant differences: a-differences in the content of TNF-α protein in the white matter compared with the control group, statistically significant at *p* < 0.05; b, differences in the content of FAS-R protein in the white matter compared to the control group, statistically significant at *p* < 0.05; c, differences in the content of FAS-L protein in the white matter compared with the control group, statistically significant at *p* < 0.05; d, differences in the content of caspase-3 protein in the white matter compared to the control group, statistically significant at *p* < 0.05; e, differences in the content of caspase-9 protein in white matter in comparison with the control group, statistically significant at *p* < 0.05; f, differences in the content of p53 protein in the white matter in comparison with the control group, statistically significant at *p* < 0.05; g, differences in the content of p-NF-kB p65 protein in white matter compared to the control group, statistically significant at *p* < 0.05; h, differences in the content of NF-kB p105 protein in white matter compared to the control group, statistically significant at *p* < 0.05.

In cortical biopsies of the epileptic focus, the content of proinflammatory TNF-α was increased (Figure 4). Similarly, cortical biopsies have shown FAS overexpression against the background of high levels of its ligand FAS-L. TNF-α content in the white matter of the temporal lobe has also increased in comparison with the values of the control group. No increase in FAS expression was found in the white matter, while the FAS-L content was significantly higher than in the control group in the gray and white matter (Figure 4 and Figure 5).

In the cortex in the epileptic focus and perifocal zone, the initiator caspase-9 expression was higher, and full-length caspase-3 was lower than in the biopsies from the control group. Caspase-3 (full-length form) and caspase-9 expression in the underlying white matter has changed similarly to values of the cortex (Figure 4 and Figure 5).

In biopsies of the cortex and white matter, proapoptotic protein p53 expression was higher than in biopsies of people without epilepsy (Figure 4 and Figure 5).

The content of S536 phosphorylated p65 subunit of NF-kB in the epileptic focus increased in the gray and white matter compared with the control group. The content of phosphorylated p105 subunit (p50 precursor) of NF-kB was significantly higher in the cortex and underlying white matter of the epileptic focus compared with the control group (Figure 4 and Figure 5).

#### 2.4.2. WB. Perifocal Zone

TNF-α expression in the perifocal zone, both in the cortex and in the white matter of the temporal lobe, exceeded its expression in biopsies of people without epilepsy. In the perifocal zone of the epileptic focus, FAS expression was increased only in the cortex. In the white matter, only an upward tendency was observed; however, no significant differences were found in comparison with people without epilepsy (Figure 4 and Figure 5).

In the cortex in the perifocal zone, caspase-9 and full-length caspase-3 expression showed the same tendency as in the epileptic focus, but less pronounced. Caspase-9 expression has increased, and full-length caspase-3 expression has decreased in the cortex and white matter in DRE patients compared with the control group (Figure 4 and Figure 5).

The proapoptotic protein p53 expression in the cortex and white matter in the perifocal zone was increased compared with people without epilepsy (Figure 4 and Figure 5).

The content of phosphorylated p65 subunit NF-kB has remained unchanged, and the content of phosphorylated p105 subunit NF-kB has increased in the cortex and white matter of the perifocal zone compared with the control group (Figure 4 and Figure 5).

### 2.5. Multiplex Biochemical Analysis of the Cytokine Profile

We have found that in plasma samples from DRE patients, the content of proinflammatory IL-1β, its natural antagonist IL-1RA, interferon IFN-γ, and anti-inflammatory IL-10 did not differ from their content in healthy people. The level of the immunoregulatory cytokine IL-2 and the chemoattractant proinflammatory IL-8 has decreased in DRE patients. The content of IL-7, which is involved in the maturation and proliferation of lymphoid cells, and proinflammatory cytokines (TNF-α, IL-4, sCD40L) was increased.

## 3. Discussion

We have previously shown the presence of apoptosis and neuroinflammation in the temporal lobe of DRE patients [9,10,21].

### 3.1. Glial Apoptosis

It is believed that astrocytes play an important, possibly central role in epileptogenesis [22]. The astrocyte membrane contains ionotropic glutamate receptors. The saturation of these receptors induces in cells the synthesis of NO (nitrogen oxide) synthase and glial acid fibrillar protein (GFAP), which protects neurons from overexcitation and death. However, the long-term presentation of epileptogenic stimuli causes a massive generation of NO in astrocytes, which has a toxic effect [23].

We have estimated the apoptosis of oligodendroglia and neurons in cortical biopsies in epilepsy (TEM). In our study, the pronounced presence of active caspase-3 in the studied biopsies from DRE patients was detected mainly in gliocytes and only in a small part of neurons (ICH) in only 20% of cases.

In the experiment, the presence of caspase-3 in the hippocampus and temporal lobe increased after an epileptic seizure in neurons [16,24] and was rarely detected in rat astrocytes [14]. It has been suggested that astrocyte apoptosis is activated during and after seizure-induced neuronal apoptosis and may contribute to neuronal death and epileptogenesis [24]. A study on rat oligodendrocyte culture has shown that oligodendrocyte apoptosis in the epileptic model was higher than in the control [15].

The main function of oligodendrocytes is to form the axon myelin sheaths. In epilepsy, the number of mature oligodendrocytes and the amount of myelin are reduced [25]. Mass demyelination of fibers in epileptic focus [12,26] causes transverse neurotransmission and generalization of nerve impulses with the simultaneous involvement of various brain regions in epileptogenesis [12]. In the rat hippocampus, the loss of myelin and oligodendrocytes begins during the acute phase and progresses in the latent and chronic phases of epileptogenesis [27].

In an experiment in mice, myelin injury caused seizures after 9–12 weeks [26]. The survival of oligodendrocytes depends on many factors, including the astrocytes condition, which secretes growth factors important for the survival of neurons, glia, and glial proliferation. For example, ciliary neurotrophic factor protects oligodendrocytes and reduces demyelination [28].

We have established that oligodendrocytes are mainly subjected to apoptosis in DRE. The oligodendrocyte loss leads to an imbalance of excitation and inhibition in the brain and provokes the formation of an epileptic focus or aggravates epilepsy severity [25]. This confirms the close interaction of all brain cellular elements and the need for their comprehensive study in pathology, in particular in epilepsy.

### 3.2. Neuronal Apoptosis and Signaling Apoptotic Pathways

In temporal lobe epilepsy, the extrinsic apoptotic pathway predominates since caspase-8 activation precedes mitochondrial dysfunction and caspase-9 activation [29]. Caspase-8 inhibitors have a more pronounced neuroprotective effect in the hippocampus than caspase-9 inhibitors [30] and prevent cytochrome C release and caspase-9 activation [16].

Caspase-8 inhibition reduces neuronal death in vitro [31] and in vivo [24]. It was found that within 1–4 h after the seizure onset, Bax clusters are formed in the outer mitochondrial membrane, which coincides with cytochrome C release and confirms the mitochondrial pathway activation [11]. After the seizure in the rat hippocampus, cytochrome C, and Apaf-1 complexes were found, which activate procaspase-9 and trigger apoptosis, and also indicate mitochondrial pathway activation [16].

In our work, an increased caspase-9 expression and a reduced caspase-3 expression in biopsies of the cortex and white matter in the epileptic focus indicates the apoptosis mediated by the mitochondrial pathway. The caspase-9 and caspase-3 content in the gray and white matter of the perifocal zone has shown the same tendency as in the epileptic focus but is less pronounced. An increased p53 content may also point to a mitochondrial pathway, which indicates DNA damage in the cells of the cortex and white matter in the epileptic focus and perifocal zone.

### 3.3. Proinflammatory Factors and Apoptosis

In our study, TNF-α and FAS-L overexpression in the cortex and white matter from DRE patients may indicate the activation of immune cells in the brain. The increased expression of proinflammatory TNF-α and FAS-L by these cells can activate the extrinsic apoptotic pathway in the cortex and white matter of the epileptic focus. In addition, FAS-L-mediated activation of immune cells in the white matter of the temporal lobe of DRE patients may lead to demyelination. In the perifocal zone, neuroinflammation also proceeds, but less intensively.

TNF-α binds to TNFR1 receptor and activates TRADD [32]. TRADD initiates competitive survival pathways caused by phosphorylation of the inhibitor protein with which NF-kB is associated. This may explain the different effects of TNF-α on apoptosis. FAS/FAS-L system initiates only the apoptosis [33].

### 3.4. Protective NF-κB Pathways

NF-κB transcription factor plays a role in chronic inflammatory diseases [31]. NF-κB homodimers p50 and p52 are gene repressors, while p65, c-Rel, p50, and p52 in any combinations are activators [34].

In our study, the content of phosphorylated subunits p65 and p105 (p50 precursor) NF-kB has increased in the cortex and white matter in the epileptic focus. Phospho p65 content in the cortex and white matter in the perifocal zone has not changed. Phospho p105 content in the cortex and white matter in the perifocal zone has increased. It can be assumed that an increased content of phospho p65 in the epileptic focus indicates the activated transcription of proinflammatory and antiapoptotic genes under TNF-α influence. This often leads not to cell death but to their survival. The increased content of phospho p105 against the background of an unchanged level of phospho p65 indicates the suppression of survival pathways in the perifocal zone. Thus, the survival mechanisms do not prevent apoptosis mediating by the extrinsic pathway in the perifocal zone.

### 3.5. Blood Cytokines

We have observed a decreased level of IL-2 in blood plasma in epilepsy. IL-2 promotes the neuron regeneration and stimulates the proliferation and differentiation of oligodendrocytes [35]. IL-2 deficiency can cause autoimmune inflammation with neuron damage [36]. The cytokines circulation (TNF-α, IL-1, IL-8) in the bloodstream usually indicates an acute phase of the body’s response to inflammation, which is regulated by proinflammatory (IFN-γ, IL-12) or anti-inflammatory (IL-10) cytokines [37]. We have found that the level of proinflammatory TNF-α, IL-7, and sCD40L increased in the blood plasma of DRE patients, and the anti-inflammatory IL-4 level has also increased compensatory. This probably reflects the systemic immune reaction associated with brain damage.

## 4. Materials and Methods

### 4.1. Study Design and Patients

The study follows a case-control design. Biopsies of 30 patients with focal DRE (24–55 years old) were studied. All patients were treated at Polenov Neurosurgical Institute, Almazov National Medical Research Centre (ANMRC), St. Petersburg, Russia (2013–2020). The work was carried out according to the principles of voluntariness and confidentiality in accordance with Federal Law “On the Basics of Health Protection of Citizens in Russian Federation” 21.11.2011 N 323-FZ, Helsinki Declaration on Human Rights and approved by the ethical committee of ANMRC. Written consents of the subjects are available. The pre-surgical stage of diagnostics was carried out according to the algorithm of the standard diagnostic complex for examining DRE patients, including clinical observation, study of neurological, neuropsychological and mental status, electrophysiological and neuroimaging investigations. The type of epileptic seizures was established in accordance with International League Against Epilepsy (2017). The patients with complex partial seizures with secondary generalization prevailed; simple partial seizures were much less.

Inclusion criteria were patients with focal DRE associated with focal cortical dysplasia; age 18 and over; epileptiform activity predominantly in the temporal lobe of the brain; established drug resistance of the disease (seizures persist after the use of two approved antiepileptic drugs basic for this form of epilepsy in the maximum tolerated doses in the form of sequential monotherapy or in combination); absence of a primary pathological substrate; confirmation of the diagnosis using invasive electroencephalographic monitoring; signed informed consent for the collection, transportation, storage and examination of biological materials.

Exclusion criteria: other structural epilepsies associated with tumors, vascular malformations, encephalitis; cerebrovascular diseases; autoimmune diseases of the brain; epilepsy with damage to other parts of the brain (frontal, parietal lobe); acute infectious, chronic inflammatory diseases.

The patients were found to have atrophy, mainly of frontal and temporal lobes, gliosis and cystic-gliosis changes in the brain, focal cortical dysplasia, and hippocampal sclerosis. The patients underwent anterotemporal resections, resections of cortical epileptic focus under the electrocorticography control. Biopsies of the cortex and white matter of the temporal lobe, obtained intraoperatively, served as material for electron microscopic, histological, immunohistochemistry (IHC), and Western blotting (WB).

The material of the comparison group for histological study, IHC (cortex and white matter of temporal lobe), was obtained during autopsies in the first 6 h after death from 6 patients who died from somatic diseases, such as acute myocardial infarction, gastric ulcer complicated by bleeding, mesenteric thrombosis, pulmonary embolism. These patients had no history of neurological disorders. Biopsy material of comparison group for histological studies, WB was obtained from 15 patients who were operated on after traumatic brain injury and had no epileptic history. These patients received reconstructive surgical treatment, such as plasty of the bones defect of the cranial vault and excision of adhesions in the area of the glio-mesodermal scar in the long-term period of the trauma (0.6–1 year). Patients with acute traumatic brain injury were not included in the study.

To detect cytokine and chemokine levels, blood plasma samples were taken from 6 DRE patients (20–32 years old). Five healthy volunteers (23–35 years old) were included in the comparison group.

The data that support the findings of this study are available on request from the corresponding author. The data are not publicly available due to privacy or ethical restrictions.

### 4.2. Histological Examination

Biopsies of the temporal lobe were fixed with 10% paraformaldehyde in 0.1 M sodium phosphate buffer, dehydrated in a standard way, and embedded in paraffin. Paraffin sections 5–7 μm thick were stained with hematoxylin and eosin (HE) by Spielmeir and Nissl, then dehydrated and mounted with neutral balsam. Sections were analyzed with a light microscope (Zeiss Axiolab, Carl Zeiss Inc., Berlin, Germany). The images at 200× and 400× magnification were used for the tissue morphological analysis.

### 4.3. IHC

Performed IHC studies of 20 patients with DRE to determine apoptosis by evaluating the presence or absence of active caspase-3. IHC reactions were performed on paraffin-embedded 5–7-μm-thick slices of the brain temporal lobe biopsies according to the standard protocol. Rabbit polyclonal antibody to active caspase-3 (1:100, PC679, Merckmillipore, Darmstadt, Germany) and EnVision polymer detection system (Dako, Santa Clara, CA, USA) were used to detect apoptotic cells. We used active caspase-3 (IHC) to demonstrate apoptosis. Full-length caspase-3 (WB), together with caspase-9, we used to study the pathways of apoptosis. To test for antibodies, staining was performed with the positive control for active caspase-3. Additionally, reactions lacking primary antibodies were done to ensure the specificity of the observed staining.

### 4.4. Transmission Electron Microscopy (TEM)

Temporal lobe biopsies were fixed in a mixture of 4% paraformaldehyde and 0.5% glutaraldehyde cooled to 4 °C (in 0.1 M cacodylate buffer, pH 7.2–7.4). Additional fixation was carried out with 1% osmium tetroxide, then the samples were dehydrated and embedded in a mixture of epoxy resins (araldites). Ultrathin sections 50–60 nm were made on ultratome (LKB-III, Sweden). The morphology was observed and recorded using TEM (FEITecnai G2Spirit BioTWIN, The Netherlands) at an accelerating voltage of 80 kV, provided by Collective Equipment Center of Sechenov Institute of Evolutionary Physiology and Biochemistry, Russian Academy of Sciences (CEC IEPB, RAS).

### 4.5. WB

The study included 30 paired biopsies of cortex and white matter from the epileptic focus and the perifocal zone of the temporal lobe from 30 DRE patients. To prevent a decrease in the number of neurons as quickly as possible and at low temperatures, a fragment weighing from 0.03 g to 0.05 g was taken from the studied biopsy specimens, a lysate buffer was added to the fragment in a ratio of 1:10, which was mixed ex tempore with protease inhibitors (Protease Inhibitor Cocktail, Sigma-Aldrich, Burlington, MA, USA) and phosphatase (PhosSTOP, Sigma-Aldrich, Burlington, MA, USA) and then homogenized. The resulting homogenate was allowed to stand in the cold for 30 min. After the destruction of the cell membrane, the samples were centrifuged for 15 min at a temperature of 4 °C with a relative centrifugal acceleration of 12,000 g to precipitate the destroyed membranes, organelles, and non-lysed cells. At the end of centrifugation, the resulting supernatant was taken, mixed with Laemmli's buffer solution, and incubated for 5 min at 95 °C. Under the action of sodium dodecyl sulfate, which is included in the buffer solution, at a high temperature, not reaching up to 100 °C, denaturation of the proteins contained in the samples occurs. As a result, an excess of negatively charged sulfonic acid residues is formed, and the intrinsic charge of the protein molecule becomes insignificant. Thus, the same ratio of negative charge to mass is achieved for any protein. After tissue pre-treatment, the total protein concentrations were determined by Bio-Rad protein assay (Bio-Rad Laboratories Inc., Hercules, CA, USA). The amount of proteins that have been separated by electrophoresis was 2 microgram/mL. We used a 10% separating gel. We used Rabbit polyclonal antibody to: NF-kB, p65 (phospho S536) ab86299, (1:500, Abcam, Waltham, MA, USA); FAS (CD95) (ab82419), TNF-α (ab9739) FAS-L(CD178) (ab186671), (1:1000, Abcam, Waltham, MA, USA); p53 (10442-1-AP), (1:1000, Proteintech, USA); Recombinant Rabbit Monoclonal Anti-NFkB p105/p50 antibody [E381] (ab32360) (1:1000, Abcam, USA); Recombinant Rabbit Monoclonal Anti-Caspase-9 antibody [E23] (ab32539) (1:1000, Abcam, Waltham, MA, USA); α/β-Tubulin Rabbit Polyclonal antibody #2148, (1:1000, Cell Signalling); Rabbit polyclonal Caspase-3 antibody (full length form) #9662 (1:1000, Cell Signalling, USA). Subsequently, the membranes were incubated with secondary anti-rabbit or anti-mouse antibodies (1:10000; Sigma-Aldrich, Burlington, MA, USA), followed by chemiluminescent detection (SuperSignal West Dura Extended Duration Substrate, Thermo Scientific, Waltham, MA, USA). The specific reaction was assessed using a gel-documenting system (ChemiDoc, BioRad, Hercules, CA, USA).

### 4.6. Multiplex Analysis

Research on Luminex MagPix (Merck, Millipore, Burlington, MA, USA) was conducted according to the manufacturer’s recommended standards and protocol on the equipment provided by CEC IEPB, RAS. All samples and standards were placed in two wells for each sample. Detection was carried out using streptavidin–phycoerythrin solution. MILLIPLEX^®^ map human cytokine/chemokine magnetic bead panel kit 96 Well Plate Assay was used.

### 4.7. Statistical Analysis

Statistical analysis was carried out by Student’s *t*-test (*p* < 0.05).

IHC. Quantitative evaluation of the results of IHC reactions (the positive IHC staining cells in temporal lobe sections) was carried out by counting in sections stained nuclei (%) per 100 cells (x200) (light microscope Zeiss Axiolab, Carl Zeiss Inc., Germany). At least 10 fields were sampled in a section. There was normal distribution. Data are presented in the format M ± m (arithmetic mean ± standard error). Statistical analysis was carried out by Microsoft Office Excel 2010 (USA), and values are expressed as mean SE for IHC.

WB. Each protein band was quantified using ImageJ 1.46 and normalized to β-tubulin expression. Statistical data processing was performed using GraphPad Prism 8.0.1. (USA). Statistical processing of the data was performed using analysis of variance (ANOVA) followed by pairwise comparison according to Tukey test because of the normal distribution of the obtained parameters. Data are presented in the format M ± m (arithmetic mean ± standard error).

Multiplex analysis. The normality test for all studied parameters was performed using Shapiro–Wilk criterion. Quantitative indicators correspond to the normal distribution and are presented in the form of an average value and a standard deviation. A comparison of quantitative data was carried out using Student’s unpaired criterion. Statistical data processing was carried out using GraphPad Prism 8.0.1. (USA).

## 5. Conclusions

Thus, we have confirmed the extrinsic and internal caspase-dependent apoptotic pathways in the epileptic brain. The detection of many apoptotic cortical neurons in biopsies with an insignificant presence of active caspase-3 may indicate a caspase-independent pathway. Intense expression of TNF-α and FAS-L in the brain and elevated levels of TNF-α, IL-7, IL-4, and sCD40L in the blood serum indicate neuroinflammation, which can cause extrinsic apoptosis in the epileptic focus. We have shown the active participation of glia apoptosis in epileptogenesis. With TEM, we have found that the main part of apoptotic glia were oligodendrogliocytes, which, in particular, explains the well-known phenomenon of myelin damage in epilepsy. The apoptosis and neuroinflammation are high in the epileptic focus and decrease in the perifocal zone.

Thus, both neuronal and oligodendroglial apoptosis in the focal DRE, together with neuroinflammation, affect the epilepsy progression, creating conditions for the development and maintenance of a pathology. Further study of this issue opens up prospects for a new therapeutic strategy.

## Figures and Tables

**Table 1:** Mean relative values (M) of caspase-3-positive glial cells in the temporal lobe brain tissue in the comparison group (without epilepsy) and in patients with epilepsy, mean error (m), number of cases (n), differences in the content of caspase-3 in the cerebral cortex compared to the comparison group statistically significant at *p* < 0.05.

	Comparison Group (n = 6)		Patients with Epilepsy (n = 20)	
	Cerebral Cortex	White Matter	Cerebral Cortex	White Matter
**Caspase-3** **(M** ± **m)**	0.33 ± 0.23	0	25.05 ± 0.70	5.94 ± 0.47

## Data Availability

The data presented in this study are available on request from the corresponding author. The data are not publicly available due to privacy reasons.

## References

[1] Bersnev V., Kravtsova S., Stepanova T., Kasumov V., Gaykova O., Odintsova G., Yaremenco N., Zagorodnicova K., Sitovskaya D., Ulitin A. (2019). Structural findings in patients with pharmacoresistant temporal epilepsy after anterior temporal lobectomy with a history of status epilepticus. Epilepsy Behav..

[2] Al-Sofyani K.A. (2021). An insight into the current understanding of status epilepticus: From concept to management. J. Neurol. Res. Int..

[3] Gidal B.E., Ferry J., Reyderman L., Piña-Garza J.E. (2021). Use of extended-release and immediate-release anti-seizure medications with a long half-life to improve adherence in epilepsy: A guide for clinicians. Epilepsy Behav..

[4] Wu J., Li J., Jing W., Tian X., Wang X. (2021). Valproic acid-induced encephalopathy: A review of clinical features, risk factors, diagnosis, and treatment. Epilepsy Behav..

[5] Trinka E., Cock H., Hesdorffer D., Rossetti A.O., Scheffer I.E., Shinnar S., Shorvon S., Lowenstein D. (2015). A definition and classification of status epilepticus—Report of the ILAE Task Force on Classification of Status Epilepticus. Epilepsia.

[6] Schmeiser B., Wagner K., Schulze-Bonhage A., Mader I., Wendling A.S., Steinhoff B.J., Prinz M., Scheiwe C., Weyerbrock A., Zentner J. (2017). Treatment of Mesiotemporal Lobe Epilepsy: Which Approach is Favorable?. Neurosurgery.

[7] Sloviter R.S. (2011). Progress on the issue of excitotoxic injury modifi cation vs. real neuroprotection; implications for post-traumatic epilepsy. Neuropharmacology.

[8] Lapchak P.A. (2012). Transcranial near-infrared laser therapy applied to promote clinical recovery in acute and chronic neurodegenerative diseases. Expert Rev. Med. Devices.

[9] Sazhina T.A., Sitovskaya D.A., Zabrodskaya Y.M., Bazhanova E.D. (2020). Functional Imbalance of Glutamate- and GABAergic Neuronal Systems in the Pathogenesis of Focal Drug-Resistant Epilepsy in Humans. Bull. Exp. Biol. Med..

[10] Sokolova T.V., Zabrodskaya Y.M., Paramonova N.M., Dobrogorskaya L.N., Kuralbaev A.K., Kasumov V.R., Sitovskaya D.A. (2017). Apoptosis of brain cells in epileptic focus at drug-resistant temporal lobe epilepsy. Transl. Med..

[11] Henshall D.C., Engel T. (2013). Contribution of apoptosis-associated signaling pathways to epileptogenesis: Lessons from Bcl-2 family knockouts. Front. Cell. Neurosci..

[12] Sitovskaya D.A., Zabrodskaya Y.M., Sokolova T.V., Kuralbaev A.K., Nezdorovina V.G., Dobrogorskaya L.N. (2020). Structural heterogeneity of epileptic foci in local drug-resistant epilepsy. Arkhiv Patol..

[13] Coulter D.A., Steinhauser C. (2015). Role of Astrocytes in Epilepsy. Cold Spring Harb. Perspect. Med..

[14] Narkilahti S., Pirttila T.J., Lukasiuk K., Tuunanen J., Pitkanen A. (2003). Expression and activation of caspase 3 following status epilepticus in the rat. Eur. J. Neurosci..

[15] Luo X., Li Z., Zhao J., Deng Y., Zhong Y., Zhang M. (2020). Fyn gene silencing reduces oligodendrocytes apoptosis through inhibiting ERK1/2 phosphorylation in epilepsy. Artif. Cells Nanomed. Biotechnol..

[16] Henshall D.C., Simon R.P. (2005). Epilepsy and apoptosis pathways. J. Cereb. Blood Flow Metab..

[17] Newton K., Strasser A. (2003). Caspases signal not only apoptosis but also antigen-induced activation in cells of the immune system. Genes Dev..

[18] Ivanova S., Polajnar M., Narbona-Perez A.J., Hernandez-Alvarez M.I., Frager P., Slobodnyuk K., Plana N., Nebreda A., Palacin M., Comis R. (2019). Regulation of death receptor signaling by the autophagy protein TP53INP2. EMBO J..

[19] Robertson J.D., Orrenius S., Zhivotovsky B. (2000). Review: Nuclear events in apoptosis. J. Struct. Biol..

[20] Shirokova A.V. (2007). Apoptosis. Signaling pathways and changes in the water and ionic balance of the cell. Cytology.

[21] Litovchenko A.V., Zabrodskaya Y.M., Sitovskaya D.A., Khuzhakhmetova L.K., Nezdorovina V.G., Bazhanova E.D. (2021). Markers of neuroinflammation and apoptosis in the temporal lobe of patients with drug-resistent epilepsy. J. Evol. Biochem. Physiol..

[22] Bedner P., Dupper A., Huttmann K., Muller J., Herde M.K., Dublin P., Deshpande T., Schramm J., Hausser U., Haas C. (2015). Astrocyte uncoupling as a cause of human temporal lobe epilepsy. Brain.

[23] Kalinichenko S.G., Dudina Y.V., Duzen I.V., Motavkin P.A. (2005). Induction of NO synthase and glial acidic fibrillary protein in astrocytes in the temporal cortex of the rat with audiogenic epileptiform reactions. Neurosci. Behav. Physiol..

[24] Engel T., Henshall D.C. (2009). Apoptosis, Bcl-2 family proteins and caspases: The ABCs of seizure-damage and epileptogenesis. Int. J. Physiol. Pathophysiol. Pharmacol..

[25] Hu X., Wang J.Y., Gu R., Qu H., Li M., Chen L., Liu R., Yuan P. (2016). The relationship between the occurrence of intractable epilepsy with glial cells and myelin sheath—an experimental study. Eur. Rev. Med. Pharmacol. Sci..

[26] Lapato A.S., Szu J.I., Hasselmann J.P.C., Khalaj A.J., Binder D.K., Tiwari-Woodruff S.K. (2017). Chronic demyelination-induced seizures. Neuroscience.

[27] Luo Y., Hu O., Zhang Q., Qian Z., Siqi H., Xiaoji T., Jiang L. (2015). Alterations in hippocampal myelin and oligodendrocyte precursor cells during epileptogenesis. Brain Res..

[28] Kıray H., Lindsay S., Hosseinzadeh S., Barnett S.C. (2016). The multifaceted role of astrocytes in regulating myelination. Exp. Neurol..

[29] Greene L.A., Liu D.X., Troy C.M., Biswas S.C. (2007). Cell cycle molecules define a pathway required for neuron death in development and disease. Biochim. Biophys. Acta.

[30] Henshall D.C., Chen J., Simon R.P. (2000). Involvement of caspase-3-like protease in the mechanism of cell death following fokally evoked limbic seizures. Neurochem..

[31] Mussbacher M., Salzmann M., Brostjan C., Bastian H., Christian S., Hannes D., Hohensinner P., Basilio J., Petzelbauer P., Assinger A. (2019). Cell Type-Specific Roles of NF-κB Linking Inflammation and Thrombosis. Front. Immunol..

[32] Lavrik I.N., Krammer P.H. (2012). Regulation of CD95/Fas signaling at the DISC. Cell Death Differ..

[33] Pérez-Figueroa E., Álvarez-Carrasco P., Ortega E., Maldonado-Bernal C. (2021). Neutrophils: Many ways to die. Front. Immunol..

[34] Prescott J.A., Mitchell J.P., Cook S.J. (2021). Inhibitory feedback control of NF-κB signalling in health and disease. Biochem. J..

[35] Fan M.Y., Low J.S., Tanimine N., Finn K.K., Priyadharshini B., Germana S.K., Kaech S., Turka L. (2018). Differential Roles of IL-2 Signaling in Developing versus Mature Tregs. Cell Rep..

[36] Huang Z., Dauer D.J., Ha G.K., Lewis M.H., Petitto J.M. (2009). Interleukin-2 deficiency-induced T cell autoimmunity in the mouse brain. Neurosci. Lett..

[37] Wojtulewicz K., Krawczyńska A., Tomaszewska-Zaremba D., Wójcik M., Herman A.P. (2020). Effect of Acute and Prolonged Inflammation on the Gene Expression of Proinflammatory Cytokines and Their Receptors in the Anterior Pituitary Gland of Ewes. Int. J. Mol. Sci..

